# Supplementary material for: Family planning uptake and its associated factors among women of reproductive age in Uganda: An insight from the Uganda Demographic and Health Survey 2016
Source: PLOS Glob Public Health. 2023 Dec 6;3(12):e0001102. doi: 10.1371/journal.pgph.0001102 (PMC10699646; doi:10.1371/journal.pgph.0001102)
Supplement: Attachment — Submitted filename: Response to Reviewers.docx [file pgph.0001102.s001.docx]

**Response to Reviewer’s Comments**

**Reviewer #1: Introduction**

**Issue 1.** Need to include socio-economical parameters in Uganda to be able to align with the findings. Example proportion of women with formal education, poverty rate, annual expenditure on health.

**Response.** Paragraph one has been improved as recommended above.

**Issue 2.** Need to include unmet need for women of reproductive age in Uganda

**Response.** Improvements have been made as recommended above.

**Issue 3.** Need to explain current situation of the known factors associated with FP uptake.

**Response.** The factors associated with FP uptake have been mentioned in the last paragraph of the introduction section.

**Issue 4.** Need to expand on the explanation of independent variables selected in this study.

**Response.** We have improved on the independent variables as indicated in the text.

**Issue 5.** Rationale for the study need to revised

**Response.** We have revised the rationale for the study.

**Methods**

**Independent variables:**

**Issue 6.** Need to define and explain how wealth index was calculated, decision making, knowledgeable about ovulatory cycle

### Response. The wealth index is a composite measure of a household's cumulative living standard. The wealth index is calculated using easy-to-collect data on a household’s ownership of selected assets, such as televisions and bicycles; materials used for housing construction; and types of water access and sanitation facilities. Generated with a statistical procedure known as principal components analysis, the wealth index places individual households on a continuous scale of relative wealth. Married women age 15-49 who are current users of family planning by who makes the decision to use family planning. Knowledge of women about the fertile period during the ovulatory cycle. The knowledge of the ovulatory cycle is the basis of the natural family planning methods.

**Issue 7.** Advised to consider gender related factors that could explain uptake of FP in Uganda. Example male engagement (accompany to FP clinics, Couple discussion on FP use). Cultural aspects like seeking prior approval from husband to use family planning. These aspects of information are usually collected in DHS

**Response.** These factors were first selected but were removed because of having a lot of missing data and outliers.

**Data Analysis**

**Issue 8.** Since the sampling procedure was multi-stage stratified cluster sampling, the authors should clearly show how did they took into account on the effect of intra and inter cluster variations.

**Response.** The design effect was used in the sample size calculation to account for the intra-cluster and inter-cluster correlation. Sampling errors was measured in terms of the standard error. Standard error was used to estimate the confidence interval. Also, sampling errors was computed in SAS, using programs developed by ICF. The programs uses Taylor linerasation method to estimate variances for the survey. The Jackknife repeated replication method was used for variance estimation.

**Results**

**Issue 9.** Result of variable ‘’ Decision maker for using contraceptive’’ was not shown despite being mentioned in the methods section under independent variables.

**Response.** Decision maker for using contraceptive was found to have a higher variance inflation factor i.e. above the cut-off value of 6 so, we decided to remove it from further analysis.

**Reviewer #2: Abstract**

**Issue 1.** First sentence: pls mention when FP use was “still low at 30%” (i.e., the year).

**Response.** The year 2020 is included as recommended above.

**Issue 2.** Is the DHS sample limited to married women of repro age or all women of repro age?

**Response.** No, it includes all women of reproductive age, 15-49 years

**Issue 3.** When you say “awareness of the availability of family planning methods” do you mean awareness of where and how to access family planning or awareness of different methods?

**Response.** We meant awareness of where the methods are and the different methods available.

**Issue 4.** Since you identified cross-sectional associations, I think you should consider toning down the implications. You conclusion sentence could say, instead, that “the study suggests that improvement in women’s education attainment, socio-economic position and awareness may help increase increases in contraceptive use in the population.”

**Response.** The conclusion has be corrected as recommended above.

**Issue 5.** Be consistent with use of “family planning” versus “FP”.

**Response.** This has been corrected as shown in the text.

**Introduction**

**Issue 6.** Authors should number the lines on each page to facilitate the review.

**Response.** We have included the number lines.

**Issue 7.** In the first para, to strengthen your argument you may wish to reference a study that links preventable maternal mortality with the prevalence of unintended pregnancies, which underscores importance of family planning as a maternal health intervention.

**Response.** We have included a reference in the first paragraph as indicated in the text.

**Issue 8.** Second para: attainment of desired family size can be achieved through natural family planning practices as well as the use of “FP methods” (which, I understood here to mean modern contraception).

**Response.** We know that natural family planning practices are also a part of family planning methods. Our study refer to the family planning methods which includes both the natural and modern methods.

**Issue 9.** Of the 1.9 billion WRA, did 1.1 billion report that they needed FP methods or that they had a desire to space or cease childbearing?

**Response.** The literature reports that they had a need for the family planning but not the desire to space or cease childbearing.

**Issue 10.** Final paragraph of page two/first para of page 3, where the explanations for the slow increase in global CPR. The authors should consider evoking some other arguments, e.g., sustained preferences for large family size. Or clarify that these explanations are supply side factors that tend to undermine the strength of FP programs.

**Response.** We have made correction as “some of the sustained preferences for the large family size are as a result of………..

**Issue 11.** Page 3 where it is written: “Therefore, universal access to FP services is an important global strategy to control fast-growing populations and improve maternal and child health.” I think the authors should be careful regarding the language that they use. Underscoring the importance of FP for “population control” has negative connotations (implies that reproductive coercion may be acceptable). They should emphasize that FP use should remain voluntary, but that access to a wide range of contraceptive methods for women’s choosing may enhance their health prospects and have wider benefits for their societies’ social and economic development.

**Response.** We have made improvements as recommended above.

**Issue 12.** “Furthermore, in 2019, over 190 million (10%) of married women were estimated to have an unmet need for FP where the prevalence was higher in Africa compared to other parts of the world (9).”
- Prevalence of what? Contraceptive use or unmet need?

**Response.** We meant prevalence of unmet need.

**Issue 13.** “This is well articulated in the sustainable development goals (SDG) 3, target 3.7 calls on countries “by 2030, to ensure universal access to sexual and reproductive health-care services, including for FP, information and education, and the integration of reproductive health into national strategies and programs”; with specifically 3.7.1 which calls for universal access to FP services to ensure healthy lives and well-being (10).”
- Should there be an‘s’ at the end of program?

**Response.** That was a typo, we meant programs.

**Issue 14.** “Despite the government efforts to reduce high fertility levels and increase uptake of FP services in Uganda, the prevalence rate is only 30% among married women which is the lowest in the East African region.”
- Please provide reference for this statement.

**Response.** We have added the citation as recommended.

**Issue 15.** “Factors contributing to the low FP rates are multi-factorial and includes…”
- Should be “and include…”

**Response.** We have corrected as “include” instead of includes.

**Issue 16.** Page 3-4: “Monitoring factors influencing the uptake of FP services is important to target scarce public resources to those with more need and enhance the progress towards achieving the global targets.”
- This sentence is confusing and should be re-written.

**Response.** We have revised the whole sentence as indicated in the text.

**Issue 17.** The review of the literature on this subject in Uganda is a bit thin. Only one study is referenced. The authors should situate their study in a more extensive review of the available literature. It does not have to be terribly long, but something more than just mentioning one study.

**Response.** We have added more FP literature in Uganda as recommended above.

**Materials and Methods**

**Issue 18.** Provide a reference for the DHS.

**Response.** The reference has been provided as recommended.

**Issue 19.** Page 5: “To generate statistics that were representative of the country as a whole in the 15 regions, the number of women surveyed in each region contributed to the size of the total sample in proportion to the size of the region.”
- I think you mean that “to generate a sample that was representation of the country.

**Response.** We meant to generate a sample that was representative of the country. This has been corrected. Thank you for the correction.

**Issue 20.** In proportion to the size (geographic?) of the region or the population size of each region?

**Response.** We meant “in proportion to the population size of each region” it is corrected as suggested.

**Issue 21.** In the study design section, I would mention that the data collection method were household surveys and women were interviewed at home.

**Response.** We have added the statement “the data collection method were household surveys and women were interviewed at home” to our study design section.

**Issue 22.** What is meant by this sentence: “Only one segment was selected for the survey with probability proportional to size, and the household listing was conducted only in the selected segment.”

**Response.** Some of the selected EAs were large in size, with more than 250 households. To minimize the task of household listing, these large EAs were segmented, and only one segment, with probability proportional to the segment size, was selected for the survey. Household listing was conducted only in the selected segment. So, a 2016 UDHS cluster is either an EA or a segment of an EA.

**Issue 23.** A question I have is whether the total number of EA per region were selected on a population proportion to size basis, or whether within all EA the numbers of households selected per region were selected on a PPS basis?

**Response.** Each region was stratified into urban and rural areas, yielding 34 sampling strata. Samples of EAs were selected independently in each stratum. The EA size is the number of residential households residing in the EA based on the 2014 Uganda Population and Housing Census. Therefore, the EAs were selected with probability proportional to the EA size.

**Issue 24.** I am a bit confused as to which aspects of the sampling strategy involved proportional allocation of sampling units to each region and which aspects were non-proportional. The first para of page 5 implies that the number of women selected per region was proportional to that regions overall contribution to the total size of the WRA population; however, here you write (on in the last para of page 6), “Due to the non-proportional allocation of the sample to different regions and their urban and rural areas, and the possible differences in response rates, sampling weights were analysed using the UDHS 2016 data to ensure that the survey results were representative at the national level as well as at the regional and sub-regional levels.”
I think that a clearer description of the sampling procedures is needed and this can reference the UDHS 2016 report where I think this is explained more clearly.

**Response.** We have improved on the description of the sampling procedures as indicated in the text.

**Issue 25.** Page 8: “The women’s questionnaire consisted of 12 sections, however, we used variables for four sections; section 1 – respondent’s background, section 3 – contraception, section 9 – fertility preferences, and section 10 – husband’s background and woman’s work”
- I think you mean women’s work.

**Response.** Yes, that was a typo but we have corrected as “women’s work”.

**Issue 26.** Data management and analysis- provide a reference that justification the VIF cut-off value of 6. This seems high to me. And I would explain procedurally how you applied the VIF cut off factor (e.g., you performed the procedure and identified all the covariates with a VIF >=6 and removed those from the model. Did you look at VIF values before or after conducting the bivariate tests of association (those estimate VIF for only the selected covariates as opposed to all of them)?

**Response.** To be more specific and accurate our VIF was below cut-off value of 4, but since we have read in some literature that equal to and less than 10 is acceptable, we decided to put us as below 6.

**Issue 27.** I would explain how the independent variables were measured (i.e., is level of education a continuous measure of number of years of schooling obtained or a categorical variable for no education, primary only, some secondary or more education). Similarly, for the wealth index, is one lowest or highest.

**Response.** We have given more meaning to our independent variables as shown in the text.

**Issue 28.** “All variables that showed a significant association of p < 0.05 at the bivariable level were further analysed at the multivariable level using a binary logistic regression. Binary logistic regression analysis was used because the data set is normally distributed and has a binary outcome. Some variables were also included in the multivariable model because these variables showed influence on the outcome variable, hence we needed to identify whether each of them had been confounded by another variable or not.”
- I found this statement confusing. It appears that you included some covariates in the multivariate model because they had a statistically significant bivariate association with the outcome, and that you included others because they “showed influence on the outcome variable” presumably this influence did not bear out in the bivariate tests but you included them anyway? I think the authors need explain more clearly how they selected covariates to include in the final model.

**Response.** The statement was misplaced in the text, we only included variables with p<0.05 in our regression analysis.

**Issue 29.** The earlier discussion on sample weights implies that the authors used a design-based modelling approach versus a model-based approach that would presumably address issues of clustering by incorporating random effects into the model. I think a more detailed discussion of how the sample weights were incorporated into the statistical modelling procedure is needed to demonstrate how the multi-level clustering effects were addressed in the analysis. To help with this, I think the authors should provide the equation for the final, multivariable model.

**Response.** The statement was misplaced in the text, so we have deleted it.

**Results of the study
Issue 30.** Description of socio-demographic characteristics of the sample is fine.
**Response.** Thank you for the compliment.

**Issue 31.** To simplify table 3 (which is too crowded and should only describe adjusted OR, in my opinion), I think that the breakdown of current FP use (yes/no) can be incorporated into Table 1 (Frequency and % can be combined in one columns with the frequency written and % included in parenthesis, and in an additional column you can put the % that are currently using an FP method).

**Response.** We have corrected our tables 1, and 3 as recommended above.

**Issue 32.** I think that this section should describe some of the results of the bivariate tests, and include a table of those results as a supplementary file. The rationale for the authors’ decision to include the retained independent variables in the multivariate tests of adjusted associations with the outcome should be clear (a sentence that reports the variables that were ultimately retained), but we do not need to see every bivariate result in the main body of the paper.

**Response.** We have removed bivarable results in the main body as recommended. However, we have retained use of family planning because the table is about compared use of current family planning methods with independent variables. The outcome variables are always compared with independent variables and that is the gist of the study.

**Issue 33.** I suspect that the models overfit. The decision to set the VIF cut off at 6 seemed high to me initially, and my review of the results reinforces this impression. In my experience (and after some cursory web searching I have done as I review the paper,) VIF <4 indicate low correlation among variables under ideal conditions, and VIF <5 is a maximum cutoff. The authors can dispute this but they need to justify the covariate selection more rigorously.

**Response.** We have responded to the above query as indicated in issue number 26 above.

**Issue 34.** Some initial impressions from Table 3:
- Be careful about including covariates that are “on the causal pathway” between another independent variable and the outcome. For example, dual household incomes are likely to drive higher levels of socio-economic attainment, which, in turn, promotes conditions in which women may access FP. I would be cautious about including both variables (women currently working and household wealth index) in the same model. Doing so can be justified in some analysis that want to estimate the different in the magnitude of effect sizes produced by models that adjust and don’t adjust for mediating factors, but this does not seem to be the case here. So the authors should map out relationships between the independent variables and outcome and think through whether adjusting for mediating variables makes sense.

**Response.** We checked all our variables for multicollinearity effect and removed those variables that had a relationship with each other so, the variables we retained have VIF of equal to and less than the value of 4.

**Issue 35.** The authors may be able to clarify this after they explain how they incorporated sample weights into the modelling procedure, but to me it is unusual to see region of residence included as a fixed effect if the sampling probabilities by region were used to develop the sample weights that were used in analysis. My intuition would be to drop the regional fixed effect.

**Response.** We have responded to this issue as indicated in line 29 above.

**Issue 36.** The inclusion of the final 10 or so covariates listed in table 3 raises some questions. Recall that this is a cross-sectional analysis. If the authors cannot demonstrate that participants’ exposure to interventions (e.g., communication interventions that disseminate FP information via radio, TV, newspapers) occurred before the occurrence of the outcome then they should be careful about using it in the model. The reason for this is reverse causality… a woman using FP might be more likely to tune into radio programs that discuss FP because they want to learn about more about the practice that they decided to uptake; a woman is more likely to get FP text messages if she is already a method user because the sender knows that this woman needs info on side effects or a reminder to come back for reinjection or resupply of other methods).
- A second reason to be concerned about included these 10 or so variables is selection bias… the selection mechanism that places women into levels of the exposure (e.g., exposure to the radio or TV program) may be very similar to the mechanisms that place them into levels of the outcome. In some instances, I think that the authors address this to some extent by adjusting for some socio-demographic characteristics (e.g., if a radio program targets youth because they are least likely to use FP, there is a common selection mechanisms for the exposure and outcome which is in part addressed by adjusting for age)… but this doesn’t address the challenge completely and introduces issues I mention about above adjusting for factors that are on the causal pathway (mediators). So, the authors should think carefully about the argument they are trying to make and decide whether including these covariates is really needed. I would encourage them to consider omitting these variables.

**Response.** Thank you for your critical thinking on the above issue. However, we have used standard variables from the DHS Program. These variables are agreed on by various technical teams selected by the USAID DHS Program including technocrats from universities and international organizations globally. Additional these variables have been adapted widely in many sub-Saharan African countries for the national survey of this kind.

**Issue 37.** On page nine the authors state that “multivariable analysis, which is a more complex analysis technique was used to understand interactions between two or more variables and also control for confounding factors.”
- I did not see any discussion of interaction (i.e., effect modification) in the methods and results.

**Response.** We have used a more realistic term which is applicable to a cross-sectional study, that is, to understand “the relationship” between two or more variables…….

**Discussion**

**Issue 38.** I think that once the methodological issues I mention above are addressed, the analysis will be simpler and yield clearer messages to reflect upon in the discussion section. As it is, the discussion is solid, but the findings are very similar to what is already well known about the determinants of FP use in the sub-Saharan African region. So, readers are likely to ask at the end of the paper “so what?”

Issue 39. On page 3, the authors mention that it is important to monitor the determinants of FP prevalence to guide how scarce public resources are allocated. This is true and I think the discussion section should elaborate on how the findings can help decision-makers use resources to strengthen the Ugandan FP program. This is implied in the discussion, but a more detailed examination of the Ugandan FP landscape and program, and how specific findings illuminate areas for improvement, or can guide the implementation of programmatic improvements, would strengthen the paper. In other words, the discussion section should do more to convince readers that this update of the determinants of FP use in Uganda is practically useful at this time.

**Response.** We have taken note of this.
