# Supplementary material for: Family planning uptake and its associated factors among women of reproductive age in Uganda: An insight from the Uganda Demographic and Health Survey 2016
Source: PLOS Glob Public Health. 2023 Dec 6;3(12):e0001102. doi: 10.1371/journal.pgph.0001102 (PMC10699646; doi:10.1371/journal.pgph.0001102)
Supplement: Attachment — Submitted filename: Response to Reviewers.docx [file pgph.0001102.s002.docx]

**Response to Reviewer’s Comments**

**Reviewer #2: Introduction**

**Issue 1.** The first sentence of the intro does not make sense grammatically.

**Response.** The sentence has been improved.

**Issue 2.** In the third sentence starting on line 39 or page, clarify what “it” is? I would provide a reference for this and possibly tone down description of the link between birth spacing and maternal death since most of the evidence on this is observation. But the point is well taken.

**Response.** We provided the correction in the previous revised manuscript.

**Issue 3.** Line 47- “Uganda aspires to become a middle-income country by 2014”… we are now in 2023… so revise the sentence accordingly.

**Response.** This has been corrected to 2025.

**Issue 4.** The assertions made between line 59 and 62 should be referenced.

**Response.** Line 59 is now referenced but line 62 already has a reference as indicated before.

**Issue 5.** Lines 63-68. Please clarify whether the literature you referenced state that 1.11 billion women expressed a need for contraceptive methods or a need to space/cease future pregnancy, and whether the 270 million had an unmet demand for contraception or an unmet desire to space/cease future pregnancy. The distinction is important because not all women that want to space/cease childbearing also want to use a contraceptive method.

**Response.** These have been corrected in the sentences.

**Issue 6.** Regarding the revision on lines 70-71 – I would give a reference that meager increase in global CPR is due to sustained preferences for large family size. My understanding is that this is really only true for sub-Saharan Africa. The factors that the authors cite as reasons for the “sustained preferences for large family size” seem more like reasons why women/families might not use contraception. Reasons to desire a large family, I would think, would have to do with the endurance of traditional cultural norms around family formation, the belief that offspring contribute more than they extract in terms of household wealth, etc.

**Response.** A reference is provided at the end as (8).

**Issue 7**. Lines 78-82. I agree with the authors point, but it could be expressed more clearly, e.g., “For this reason, numerous scholars have pointed out that promoting voluntary access to a wide variety of contraceptive methods for women is an important component of countries’ strategies to advance social and economic development.”

**Response.** The sentences have been improved as recommended.

**Issue 8.** I think the intro has all the needed details, but its structure is a bit confusing. It starts by pointing out the importance of family planning as a health intervention, then describes the profile of Uganda, and then circles back to explain why FP is important. I think the paper would benefit from a slight reorganization of the intro so that it describes (1) the health and demographic background of Uganda as relevant, (2) how increasing FP use can accelerate achievement of major health and development goals in the country, and (3) what is known from relatively recent research on the factors that influence practice of family planning in the country and justification for the paper.

**Response.** Sir, we have structured the background according to your recommendation in the first revision. We feel your first recommendation serves the purpose.

**Methods**

**Materials and Methods**

**Issue 10.** Line 228 - Why the decision to measure age categorically rather than continuously in the final model? If you are able to show that positive differences in women’s age are associated with their odds of FP use when age is modelled continuously, I would do it that way.

**Response.** Age was already categorized in the dataset we used.

**Issue 11.** Line 257-259: Did you calculate the VIFs for all variables that proved significantly associated with outcome in the bivariable analysis and then exclude those with a VIF > 4; or did you calculate the VIF first for all variables before the bivariate analysis and conduct the bivariate analysis for all covariates with a VIF < 4? Intuitively , the best way seems to be removing variables based on VIF after determining which ones are significantly associated with the outcome in bivariate tests (which is what I think the authors did but its not clear).

**Response.** We removed variables during bivariate tests.

**Issue 12.** The data have a hierarchical structure (i.e., individual women are nested in households, households nested in enumeration areas, and enumeration areas nested in stratum), however, it is not clear how the authors accounted for this in the modelling strategy? Doing so is quite crucial since failing to account for clustering in analysis can result in biased inference which generally results in Type 1 error. The authors need to address this. Depending on the information available to them they can use a model- or design-based approach for this (the easier would be a model-based approach that incorporates random effects, I think)… but given the multiple levels of clustering I would imagine the authors should compare the different ways to fit the mixed-effects model and select the model with the highest “goodness of fit”.

**Response.** At the design stage, during data collection and data presentation, type 1 errors and other systematic errors were catered for. This is secondary data and a national dataset that already underwent cleaning. Also, refer to our earlier response to this issue.

**Results of the study**

**Issue 13.** Table 2 is a bit confusing with regards to “pattern of family planning use”. Cannot a woman have used a method before AND after last birth? Similarly, a current user could also be a woman that has used it since last birth? Unless this finding is really important, you might consider removing it from the table.

**Response.** We have used standard variables used by the UDHS, which are useful for the Uganda setting.

**Issue 14.** In table 2, under current use by methods, it is written that 458 women are currently using a traditional method and below under “intention to use contraceptive” it is written that 504 women are using a traditional method. I think it might be more useful to present the breakdown of users/non-users in terms of their desire to space and/or limit future pregnancy, i.e. (1) women that desire to space or limit that are currently using a method, (2) women that do not have a birth timing preference, but are using a method anyway, (3) women that want a child soon and are using a method, (4) women with a desire to space or limit that are not using a method, (5) women that have no preference and are not using a method, (6) women that want a child soon and are not using a method.

**Response.** All variables are according to the categories in the UDHS dataset so, we cannot invent the wheel (variables that are not within the dataset).

**Issue 15.** Line 310 – Because the data are cross-sectional, it is hard to tell whether some of the independent variables are truly determinants, but you can call them “factors associated with family planning uptake”.

**Response.** We have taken care of this.

**Issue 16.** Lines 323-326 refer to the “goodness of fit of the model”. I think this really only makes sense if you describe what other models you fit and compared when you were determining which to report as the final model. I am unfamiliar with the use of Pearson chi-sq test for assessing goodness of fit, however with logistic mixed effects models (which is what I think the authors should use given the multi-level nature of the data), I think the authors should compare the AIC/BIC or loglikelihood values associated with models with alternative specifications for dealing with clustering and chose the most appropriate one that way. This can be described in the methods section, rather than in the results section.

**Response.** We are conversant with the use of Chi-square as a goodness of fit in relation to the simultaneous modeling technique and we believe we chose the best method of analysis according to the outcome variable.

**Issue 17.** The variable “can a woman be pregnancy after birth” needs to be described more… When after birth? Are you referring to women’s awareness of their fertility postpartum (i.e., do women know WHEN they return to being fertile after childbearing). And what does the finding imply?

**Response.** This means whether a woman can be pregnant immediately after giving birth. Again, we used all variables as they were written in the UDHS.

**Issue 18.** The authors chose to retain a lot of variables in the final model. My advice is (1) the authors should frame this by stating the hypothesis that multi-level sets of factors affect the outcome, and these levels reflect women’s individual level characteristics (e.g., age, education), household/spousal characteristics (e.g., wealth, husband education), geographic context (e.g., rural/urban, region), and their exposure to programs (e.g., heard of FP on TV, in newspaper); (2) then, the authors should fit different versions of the model that they selected based on comparing AIC and BIC values for versions that adjust for (a) individual, household and geographic covariates, and (b) individual, household, geographic and programmatic covariates. AIC and BIC values reported for each model should be included in the analysis.

**Response.** According to our knowledge multivariable regression using simultaneous modeling technique uses up to 25 variables. You can check this in the SPSS Software under binary regression analysis.

**Discussion and Conclusion**

**Issue 19.** Since the paper already has an introduction, I do not think that the sub-section under the discussion should also be called an introduction.

**Response.** This is corrected as recommended.

**Issue 20.** The findings seem to be that in 2016, women’s age, educational attainment and household wealth status were associated with their odds of currently using contraception. I think the discussion of these findings need to be framed in the context of an ongoing policy or programmatic deliberating occurring in Uganda to help readers understand why these findings are important. On the surface, these are relatively old data that tell us what we already know.

**Response.** We believe these findings are still useful to the Ugandan context besides, the methods used and recommendations/conclusions are very important as well. We believe the academia, health providers, and policymakers would benefit from it.

**Issue 21.** What do the authors think about the litany of null effects for the programmatic covariates (except “heard about FP on radio)? Based on the finding, should the discussion urge programs to do a better job at implementation? Since its not possible to ascertain whether the programmatic exposures occurred before or after the outcome of interest, does it make sense to show these results?

**Response.** We believe it is useful because we have so many people owning radios in Uganda and this could be an opportunity to disseminate FP services and findings like this one.

**Issue 22.** Line 410-412. The authors say that their analysis reveals that interventions that bring FP closer to women would increase uptake more than static services, but I cannot locate this finding in the analysis or discussion. I do not understand why the authors state this as an implication.

**Response.** This has been deleted.

**Issue 23.** Lines 416-427. A major limitation is that the data are cross-sectional and the authors could not establish temporality between participants exposure to some of the independent variables (i.e., program exposure) and the outcome. The data are also 7 years old so the implications of the analysis may be too dated.

**Response.** This has been considered. However, this manuscript was first submitted before the duplication of the new UDHS 2022 key findings. Besides, the main report is not yet published and the dataset is not available in the DHS Program.
